# Aldosterone-targeted therapies: early implementation in resistant hypertension and chronic kidney disease

**DOI:** 10.1093/eurheartj/ehaf225

**Published:** 2025-04-11

**Authors:** Masatake Kobayashi, Bertram Pitt, João Pedro Ferreira, Patrick Rossignol, Nicolas Girerd, Faiez Zannad

**Affiliations:** Université de Lorraine, INSERM, Centre d'Investigations Cliniques 1433, CHRU de Nancy, Inserm 1116 and INI-CRCT (Cardiovascular and Renal Clinical Trialists) F-CRIN Network, Nancy, France; Department of Cardiology, Tokyo Medical University, Tokyo, Japan; University of Michigan School of Medicine, Ann Arbor, MI, USA; Université de Lorraine, INSERM, Centre d'Investigations Cliniques 1433, CHRU de Nancy, Inserm 1116 and INI-CRCT (Cardiovascular and Renal Clinical Trialists) F-CRIN Network, Nancy, France; Department of Surgery and Physiology, Faculty of Medicine of the University of Porto, Cardiovascular Research and Development Center, Porto, Portugal; Université de Lorraine, INSERM, Centre d'Investigations Cliniques 1433, CHRU de Nancy, Inserm 1116 and INI-CRCT (Cardiovascular and Renal Clinical Trialists) F-CRIN Network, Nancy, France; ALTIR, Nancy, France; Medical Specialties and Nephrology Dialysis Departments, Monaco Princess Grace Hospital and Monaco Private Hemodialysis Centre, Monaco, Monaco; Université de Lorraine, INSERM, Centre d'Investigations Cliniques 1433, CHRU de Nancy, Inserm 1116 and INI-CRCT (Cardiovascular and Renal Clinical Trialists) F-CRIN Network, Nancy, France; Université de Lorraine, INSERM, Centre d'Investigations Cliniques 1433, CHRU de Nancy, Inserm 1116 and INI-CRCT (Cardiovascular and Renal Clinical Trialists) F-CRIN Network, Nancy, France

**Keywords:** Mineralocorticoid receptor antagonist, Aldosterone synthase inhibitor, Treatment-resistant hypertension, Chronic kidney disease, Heart failure, Aldosterone

## Abstract

Treatment-resistant hypertension (TRH) often coexists with chronic kidney disease (CKD), and the presence of both conditions increases the risk of adverse cardiovascular outcomes. Patients with TRH and CKD exhibit enhanced aldosterone and mineralocorticoid receptor expression, which promote inflammation and fibrosis in cardiac and renal tissues, contributing to the development and progression of cardiorenal diseases. Both achieving optimal blood pressure (BP) control and mitigating the risk of aldosterone-related adverse events are cornerstones in the management of patients with TRH and CKD. Mineralocorticoid receptor antagonists (MRAs) are recommended for the treatment of TRH. To date, the efficacy has been investigated in populations with mostly normal renal function. However, the potential risk of hyperkalaemia limits the use of MRAs, particularly in patients with CKD. Non-steroidal MRAs and sodium glucose cotransporter-2 inhibitors have slowed renal function decline and shown cardiorenal benefits. Additionally, aldosterone synthase inhibitors may emerge as a therapeutic option for patients with TRH. Clinical trials for TRH primarily centred on assessing BP-lowering effects; however, merely lowering BP might not be a sufficient target to prevent a risk of cardiorenal disease progression. This paper presents evidence and potential benefits of aldosterone-targeted therapy in the treatment of TRH and CKD and re-consider the treatment strategies in clinical practice and trial design.

## Graphical Abstract

**Graphical Abstract:**
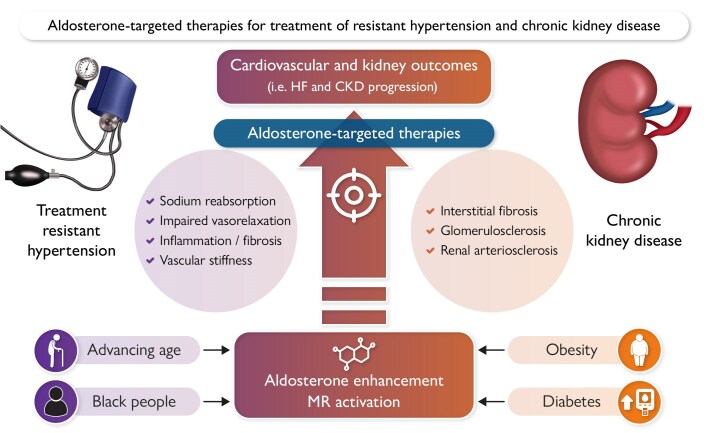

## Introduction

Patients with treatment-resistant hypertension (TRH) often have comorbid chronic kidney disease (CKD).^1,2^ The concurrent presence of both diseases synergistically and bidirectionally contributes to disease progression and an increased risk of adverse cardiovascular (CV) and renal outcomes.^3,4^

Compelling evidence suggests that aldosterone over-production is an important substrate for TRH.^5,6^ Excessive aldosterone may promote inflammation and fibrosis in myocardial, vascular, and renal tissues, thereby leading to the progression of TRH and CKD severity.^7,8^ While optimized blood pressure (BP) control is crucial for reducing cardiorenal risk, excessive aldosterone can increase the risk regardless of BP levels.^9,10^ Both achieving BP control and mitigating the risk of aldosterone-associated cardiorenal outcomes should be the focus when managing patients with TRH and CKD.

The guidelines recommend mineralocorticoid receptor antagonists (MRAs) as a fourth-line treatment for TRH.^2^ This is based on trial data showing that spironolactone vs alternative treatments significantly lowered BP,^11^ especially in patients with a biochemical profile indicative of more enhanced aldosterone.^12^ Therefore, non-steroidal MRAs and an aldosterone synthase inhibitor (ASI) may be promising options to target aldosterone activations for TRH and/or CKD.

The potential risk of hyperkalaemia limits the broader use of MRA therapy, particularly in patients with CKD. Potassium binders and diuretics are often used to manage the risk of MRA-associated hyperkalaemia, while sodium–glucose co-transporter 2 inhibitors (SGLT2is) not only mitigate cardiorenal risks but may also reduce the likelihood of MRA-associated hyperkalaemia.^13–15^

This paper presents available evidence and explores the potential advantages of aldosterone-targeted therapy in managing TRH and CKD, while proposing a re-evaluation of treatment strategies within the realms of clinical practice, guidelines, and trial design.

## Treatment-resistant hypertension and rate of chronic kidney disease coexistence

Overall, patients with moderate-to-advanced CKD had a two- to three-fold higher risk of TRH compared with those without CKD.^16,17^ In a study of >10 000 hypertensive patients, CKD progression, defined by a lower estimated glomerular filtration rate (eGFR) or a higher urine albumin-to-creatinine ratio (UACR), was associated with higher TRH prevalence.^16^ Among 4901 participants with CKD on antihypertensive medication from Germany, 38% were categorized as having TRH, which was independently associated with lower eGFR and higher UACR in a multivariable model.^18^ In the CRIC study including 3612 patients with CKD (TRH prevalence 40%), a 5 mL/min/1.73 m^2^ decrease in eGFR was associated with 4% higher odds of having TRH.^19^ Among 3664 Japanese patients with CKD, patients with TRH (16%) were more likely to have low eGFR.^20^ In the ALLHAT trial including 33 357 participants with hypertension and an additional CV risk factor, a serum creatinine level of >1.5 mg/dL was a predictor of failure to achieve BP control, defined as <140/90 mmHg.^21^ A meta-analysis of 91 studies with >3 million patients on antihypertensives showed TRH prevalence to be 10.3% overall and 22.9% in those with CKD.^22^ Observational studies on the associations between TRH and CKD are summarized in *Table 1*.

**Table 1.** Observational data on the association between treatment-resistant hypertension and chronic kidney disease

Study (year)	Number of patients	Population	TRH definition	TRH prevalence	Main findings
**The Kaiser Permanente Southern California health system, 2012** ^(23)^	205,750	Hypertensive patients	TRH was defined as BP ≥140/90 mm Hg on ≥3 antihypertensives	1.9% developed TRH within a median of 1.5 years	During a median follow-up of 3.8 years (2.6–4.8), patients with TRH were more likely to experience the composite outcomes of death, MI, HF, stroke, or CKD, compared with those with non-TRH (18.0% vs 13.5%, *P* = 0.001; HR, 1.54; 95% CI, 1.40–1.69).
**The Kaiser Permanente Southern California health system, 2015** ^(24)^	470,386	Hypertensive patients	TRH was categorized as controlled BP, controlled on ≥4 antihypertensives, or uncontrolled BP, uncontrolled on ≥3 antihypertensives	12.8%	From a 2–5-year follow-up period, patients with TRH had a higher risk of developing ESKD, compared with those with non-TRH (adjusted HR, 95% CI = 1.32, 1.27–1.37). The adjusted risk of ESKD was 20% higher in uncontrolled TRH vs controlled TRH.
**The REGARDS study, 2013** ^(16)^	10,700	Patients with CKD (eGFR 30–60) who were taking one or more classes of antihypertensive medication.	TRH was defined as BP ≥140/90 mm Hg on ≥3 antihypertensives, or use of ≥4 antihypertensives regardless of BP levels	17.9%	The prevalence of TRH was 15.8%, 24.9%, and 33.4% for patients with eGFR ≥60, 45–59, and <45 mL/min/1.73 m^2^, respectively, and 12.1%, 20.8%, 27.7%, and 48.3% for UACR<10, 10–29, 30–299, and ≥300 mg/g, respectively.
**The REGARDS study, 2014** ^(25)^	9,974	Patients on antihypertensive medication without ESKD at baseline	TRH was defined as BP ≥140/90 mm Hg on ≥3 antihypertensives, or use of ≥4 antihypertensives regardless of BP levels	21.5%	During a median follow-up of 6.4 years, the incidence of ESKD per 1,000 person-years was higher in patients with TRH vs non-TRH [8.86 (7.35–10.68) vs 0.88 (0.65–1.19)]. Patients with TRH were more likely to experience the development of ESKD, compared with those with non-TRH (adjusted HR, 95% CI = 6.32, 4.30–9.30).
**The ALLHAT trial, 2014** ^(26)^	14,684	Hypertensive patients at least one other coronary heart disease risk factor	TRH was defined as BP ≥140/90 mm Hg on ≥3 antihypertensives, or use of ≥4 antihypertensives with BP at goal during the study visit. Use of a diuretic was not required to meet the definition of TRH.	12.7%	During a median follow-up of 4.9 years, patients with TRH were more likely to experience the development of ESKD, compared with those with non-TRH, independent of baseline eGFR (adjusted HR, 95% CI = 2.11, 1.20–3.70).
**The Chronic Renal Insufficiency Cohort, 2015** ^(17)^	3,367	Hypertensive patients with CKD (eGFR 20–70)	TRH was defined as BP ≥140/90 mm Hg on ≥3 antihypertensives, or use of ≥4 antihypertensives with BP at goal at baseline visit	40.4%	During a median follow-up of 5.0 years, patients with TRH had a higher risk of developing renal events (i.e. a 50% decline in eGFR and/or ESKD) compared with those with non-TRH (adjusted HR, 95% CI = 1.28, 1.11–1.46).
**Sub-analysis of Multifactorial Approach and Superior Treatment Efficacy in Renal Patients With the Aid of Nurse Practitioners, 2015** ^(27)^	788	Patients with CKD (eGFR 20–70)	TRH was defined as a BP ≥130/80 mm Hg despite treatment with ≥3 antihypertensive drugs, including a diuretic or treatment with ≥4 antihypertensive drugs.	34.0% (office blood pressure) and 32.0% (automated measurements)	During a median follow-up of 5.3 years, 27% of patients with TRH reached ESKD. TRH increased the risk of developing ESKD by 2.3-fold (95% CI 1.4–3.7).
**The Fukuoka Kidney disease Registry, 2022** ^(20)^	3,664	Patients with CKD (eGFR 20–70)	TRH was defined as BP ≥140/90 mm Hg on ≥3 antihypertensives, or use of ≥4 antihypertensives regardless of BP levels	16.0%	The prevalence of TRH was significantly associated with BMI, decreased eGFR (1.87, 1.57–2.23), as well as age, diabetes mellitus, and chronic heart disease.
**The German Chronic Kidney Disease (GCKD) study, 2023** ^(18)^	4,901	Patients with CKD (eGFR 30–60 or overt proteinuria of an eGFR >60) on antihypertensive medication	TRH was defined as BP ≥140/90 mm Hg on ≥3 antihypertensives	38.0%	The higher prevalence of TRH was independently associated with male sex, older age, lower eGFR, higher BMI, higher UACR, and the presence of diabetes mellitus. During a 6-year follow-up period, patients with TRH had a higher risk for developing ESKD compared with those with non-TRH (adjusted HR, 95% CI = 1.50, 1.17–1.92).

BMI, body mass index; BP, blood pressure; CI, confidence interval; CKD, chronic kidney disease; eGFR, estimated glomerular filtration rate; ESKD, end-stage kidney disease; HF, heart failure; HR, hazard ratio; MI, myocardial infarction; TRH, treatment-resistant hypertension; UACR, urine albumin-to-creatinine ratio.

## Treatment-resistant hypertension and chronic kidney disease progression

Although TRH prevalence in the context of CKD has been extensively reported, patients with TRH also have a higher risk of developing end-stage kidney disease (ESKD), compared with those with non-TRH, independent of baseline renal function.^25^ Overall, the risk of developing ESKD was assessed over a ∼5-year follow-up period between patients with TRH and non-TRH, and the risk was ∼two-fold higher in those with TRH vs non-TRH.^17,18,24,27^ In the ALLHAT trial (*n* = 14 684), patients with TRH (12.7%) were more likely to develop ESKD, compared with those with non-TRH during a median follow-up of 4.9 years.^26^ In the CRIC study (*n* = 3367), patients with TRH (40.4%) had a higher risk of developing renal outcomes (i.e. ESKD and a 50% decrease in eGFR) during a median follow-up of 5.0 years.^17^ In database from a healthcare system in Southern California including >470 000 hypertensive patients, TRH prevalence was associated with a higher risk of developing ESKD compared with non-TRH, and the future ESKD risk was lower in patients with controlled TRH (BP <140/90 mmHg on ≥4 antihypertensive medications) compared with uncontrolled TRH (BP ≥140/90 mmHg on ≥3 antihypertensive medications) [hazard ratio (HR), 95% confidence interval (CI) = 0.80, 0.75–0.85].^24^ Observational studies on the associations between TRH and the risk of ESKD are summarized in *Table 1*.

## Aldosterone and mineralocorticoid receptor activation

### The role as blood pressure regulation, endothelial dysfunction, and arterial stiffness

Aldosterone is mainly produced by the adrenal glomerulosa in response to angiotensin II, adrenocorticotropic hormone, and increased serum potassium, and it binds to MR in the distal convoluted tubule of the kidney.^28,29^ Classically, aldosterone increases sodium reabsorption in the distal nephron to maintain sodium balance via activation of the apical epithelial sodium channel (ENaC) and the basolateral Na/K-ATPase pump.^30^ Additionally, aldosterone activates serum- and glucocorticoid-inducible kinase-1, which enhances ENaC activity and suppresses Nedd4-2. This suppression decreases the ubiquitination and degradation of ENaC channels^31^; thus, aldosterone activation results in a greater number of ENaC channels, leading to increased sodium reabsorption and elevated BP.

Aldosterone also impairs endothelial function by inhibiting the activity of endothelial nitric oxide synthase, increasing reactive oxygen species, and reducing nitric oxide bioavailability.^32,33^ Additionally, by increasing vascular myogenic tone and the expression of pro-inflammatory cytokines in vascular cells, aldosterone promotes vascular smooth muscle cell proliferation and collagen deposition in arterial walls, contributing to the progression of arterial stiffness.^34,35^

### Profibrosis and pro-inflammation in kidneys

The role of aldosterone in inflammation, tissue remodelling, and fibrosis in the kidney has been well documented.^8,30^ Aldosterone exerts deleterious effects in the kidneys even in the absence of angiotensin II,^36,37^ whereas the MR expression in the kidney (i.e. podocytes, endothelial cells, mesangial cells, and tubular epithelial cells) supports the role of aldosterone in renal injury.^38,39^ The MRs in the kidney are enhanced in response to aldosterone activation, leading to increases in plasminogen activator inhibitor-1, transforming growth factor-beta (TGF-β), and interleukin-6 as mediators of fibrosis.^40,41^ Plasminogen activator inhibitor-1 increases extracellular matrix accumulation and fibrosis in the kidney,^42^ while TGF-β promotes fibrosis by stimulating the transformation of cells into fibroblasts.^43^ Additionally, the expression of MRs in podocytes or endothelial cells directly caused glomerulosclerosis or renal arteriosclerosis, respectively.^44,45^

Mineralocorticoid receptor can also be activated by cortisol.^46^ Under normal circumstances, aldosterone is co-expressed with the enzyme 11-beta-hydroxysteroid dehydrogenase-2 (11β-HSD2), which inactivates cortisol in the kidneys.^47^ However, under certain circumstances such as visceral obesity, hypertension, heart failure (HF), and/or CKD, cortisol may become available to activate the MR due to the down-regulation of 11β-HSD2, potentially leading to inflammation and fibrosis in the kidneys.^40,48,49^

The renal effects of aldosterone expression or MR over-activation mirrored the results of a clinical study that included 3680 patients with CKD. Over a median follow-up of 9.6 years, each doubling of serum aldosterone increased CKD progression risk by 11% (i.e. 50% decline in eGFR, chronic dialysis, or kidney transplant), independent of baseline eGFR and 24-h urine protein.^50^

### Renin-dependent aldosteronism and primary aldosteronism

**Figure 1:**
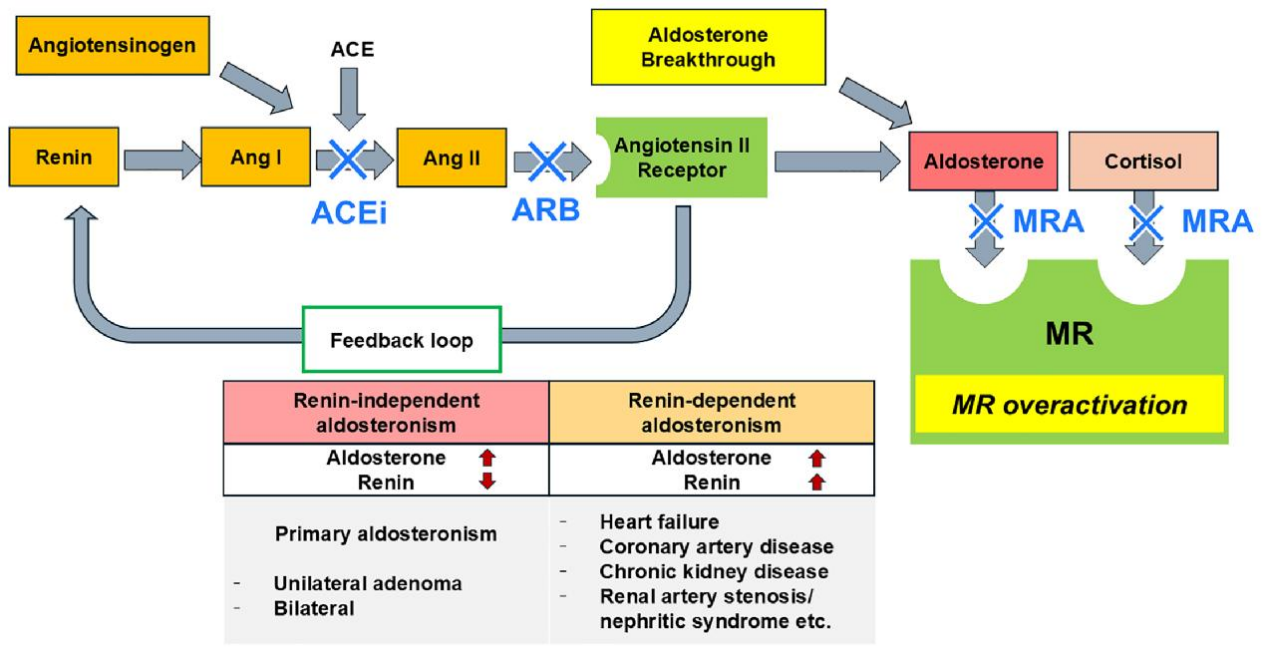
Renin-independent and dependent aldosteronism. Aldosterone is enhanced in cardiorenal diseases through renin-dependent aldosteronism and often rebounds over the long term even after initiating an inhibitor of renin–angiotensin aldosterone system (aldosterone breakthrough). Cortisol also activates the MRs, particularly in cardiomyocytes and podocytes. Additionally, the MRs are activated and overexpressed, independent from aldosterone levels in cardiorenal diseases. ACE, angiotensin-converting enzyme; Ang I, angiotensin I; Ang II, angiotensin II; ACEi, angiotensin-converting enzyme inhibitor; ARB, angiotensin receptor blocker; MRA, mineralocorticoid receptor antagonist; MR, mineralocorticoid receptor

Several cardiorenal diseases (i.e. HF, coronary artery disease, and CKD) enhance aldosterone through renin-dependent aldosteronism (*Figure 1*). These diseases were classically managed with angiotensin-converting enzyme inhibitor or angiotensin receptor blocker (ACEi/ARB), which suppresses aldosterone over the short term. However, aldosterone often rebounds over the long term (>6 months), a phenomenon known as aldosterone breakthrough.^51^ In addition, the hyper-aldosterone state in cardiorenal diseases is not only related to excess aldosterone levels. It is mainly driven by an increased aldosterone ligand-dependent activation of MRs.^52^ The MRs are activated and overexpressed, independent from aldosterone levels in cardiorenal diseases.^52–54^ Also, cortisol is the other natural MR ligand, which may also activate the MRs in pathological states (*Figure 1*). Therefore, even for individuals with renin-dependent aldosteronism, MRA therapy is essential for mitigating the insufficient and short-lived control of aldosterone excess by ACEi/ARBs, blocking the over-activated receptor, shielding it from both aldosterone and cortisol effects, and improving prognosis as proved by pivotal clinical trials.^55–60^

In contrast, primary aldosterone is defined as autonomous or non-suppressible renin-independent aldosterone production and affects around 20% of patients with TRH.^61^ Guideline-recommended screening for primary aldosteronism remains underutilized (<2%) with little change over 2 decades.^62^ In masked uncontrolled hypertension, aldosterone secretion increased only when measured outside the clinic.^63^ Primary aldosteronism causes glomerular hyperfiltration, which progressively accelerates declines in glomerular filtration rate and promotes albuminuria.^64–66^ A meta-analysis of 46 studies showed that primary aldosteronism (*n* = 6056) was associated with a higher risk of albuminuria excretion than arterial hypertension (*n* = 9733) independent of BP levels over 8.8 years.^67^ Additionally, from a meta-analysis of 31 studies, primary aldosteronism (*n* = 3838) was also associated with a higher risk of coronary artery disease [odd ratio (OR) = 1.77], atrial fibrillation (OR = 3.52), HF (OR = 2.05), and left ventricular hypertrophy (OR = 2.29) compared with essential hypertension (*n* = 9284) over 8.8 years.^68^

Mineralocorticoid receptor antagonist therapy is used for primary aldosteronism, while surgical adrenalectomy is recommended for unilateral cases, given its low operative risk.^69,70^ Whether clinical benefits of surgical adrenalectomy outweigh MRA therapy remains unclear, though both significantly reduced CV and renal event risks.^65,71^ In a cohort study of 303 patients with primary aldosteronism treated with MRA therapy, larger decline in eGFR after MRA initiation was associated with smaller long-term decline in eGFR over 4.5 years, suggesting that MRA therapy may reverse glomerular hyperfiltration and uncover the underlying renal function in primary aldosteronism.^72^

## Shared risk factors for treatment-resistant hypertension and chronic kidney disease and their effects on aldosterone and mineralocorticoid receptor activation

Aging, race (particularly Black people), obesity, and diabetes are common clinical factors in both TRH and CKD. While causal relationships between some of these factors and TRH/CKD remain unclear, they are associated with aldosterone enhancement and/or MR expression.^73,74^

### Advancing age

Advancing age is a prominent risk factor for both TRH and CKD. Among 205 750 hypertensive patients, 1.9% of those who developed TRH within a median of 1.5 years were older than those who did not.^23^ Similarly, the prevalence and incidence of CKD were higher in older people.^75^ Although serum aldosterone was generally within the normal range in older patients, they exhibited a higher likelihood of MR over-expression compared with younger counterparts.^76^ Furthermore, in rat models, the removal of MR in vascular smooth muscle cells mitigated age-related vascular stiffening and fibrosis.^77^

### Black people

In the USA, TRH is more common in Black people than in White people with hypertension.^78,79^ Likewise, Black people have a higher risk of developing CKD than their White counterparts.^80^ Although socioeconomic and genetic factors (e.g. the apolipoprotein L1 gene) substantially affect the prevalence of hypertension and/or CKD,^81–83^ they may also confer a genetically determined predisposition to aldosterone activation.^84^ This activation leads to overactivity of the ENaC, enhancing sodium reabsorption in the renal tubules and suppressing plasma renin activity.^84^ In a study of 912 African Americans, lower plasma renin activity and higher aldosterone-to-renin ratio were associated with elevated office and ambulatory BP, highlighting renin-independent aldosteronism as a key feature in this population.^85^

### Obesity

Obesity is a causal risk factor for TRH and is ∼two-fold risk factor for TRH.^86,87^ Excessive weight gain increases BP, particularly in people with visceral obesity, while higher body mass index was associated with greater CKD prevalence and incidence.^88,89^ Obese patients may experience increased aldosterone secretion or MR expression, triggered by activation of renin–angiotensin system and leptin from adipocytes.^7,90^ Additionally, adipocyte-derived cytokines express the guanosine triphosphate-binding protein, Rac1, which has the potential to activate the MR.^39,91^

### Diabetes

Diabetes is associated with an increased prevalence of TRH and CKD. Using data from the National Health and Nutrition Examination Survey, among hypertensive patients, 8.9% of patients were diagnosed with TRH. Those with TRH had a higher prevalence of diabetes compared with those without.^92^ Several prior studies consistently showed a higher prevalence of diabetes among patients with TRH compared with those without^93,94^ whereas diabetes is the most common cause of developing and progressing CKD worldwide.^95^ In patients with diabetes and/or CKD, hyperglycaemia induces protein kinase C signalling, leading to over-activation of aldosterone and up-regulation of MR expression.^96–98^

## Comparative pharmacology of mineralocorticoid receptor antagonists and mineralocorticoid receptor antagonists under development

### Steroidal mineralocorticoid receptor antagonists

Spironolactone, as the first generation of MRAs, was initially introduced as a diuretic for managing primary aldosteronism and essential hypertension.^99^ Eplerenone as the second generation of MRAs was designed with higher specificity, lower affinity, and potency for binding to the MR compared with spironolactone, resulting in marginally fewer hormonal adverse effects.^100^ Other pharmacologic difference is that spironolactone is a pro-drug and is converted to canrenone, a biologically active metabolite with a longer half-life of >20 h, which can accumulate over time, while eplerenone has a shorter half-life of 4–6 h.^74^ The difference may potentially account for stronger BP-lowering effect of spironolactone than eplerenone, although eplerenone has shown comparable CV benefits to spironolactone in HF with reduced ejection fraction (HFrEF).^57–59^

### Non-steroidal mineralocorticoid receptor antagonists

Non-steroidal MRAs inhibit MR activity while possessing less specific tissue distribution to the kidney, higher affinity, and greater specificity compared with steroidal MRAs, which may be linked to a lower risk of hormonal side effects.^101^ In addition, non-steroidal MRAs have different co-factor recruitment patterns after MR binding compared with steroidal MRAs,^101,102^ which might differentially affect gene expression, resulting in more potent anti-inflammatory and anti-fibrotic effects of non-steroidal MRAs even at comparable natriuretic doses.^101^

The half-life of non-steroidal MRAs may characterize their capacity to lower BP. For example, finerenone has a relatively low BP-lowering effect due to very short half-life (∼2.5 h) and low lipophilic properties, which implies a reduced likelihood of finerenone crossing the blood–brain barrier and antagonizing the MR in the hypothalamus, a key factor in BP regulation.^103^ Ocedurenone has a long half-life of ∼60 h and is not affected by dialysis, which was expected to show strong BP-lowering effects.^104^ In preclinical models, ocedurenone was more effective than eplerenone in lowering BP, attenuating hypertension development, and reducing 24-h urinary albumin excretion.^105^ Esaxerenone also has a relatively long plasma half-life of 20–30 h.^106^ In a model of hypertensive rats, esaxerenone reduced proteinuria, renal hypertrophy, left ventricular hypertrophy, and brain natriuretic peptide levels greater than spironolactone despite similar BP-lowering effects.^107^

## Blood pressure–lowering effects of mineralocorticoid receptor antagonists in clinical trials

### Spironolactone

Contemporary guidelines for TRH derive from the PATHWAY-2 trial (*Table 2*). Spironolactone 25–50 mg/day reduced systolic BP by 12.8 mmHg over 12 weeks, surpassing the effects of bisoprolol (5–10 mg/day) and modified-release doxazosin (4–8 mg/day).^11^ In the mechanistic sub-study, patients with lower plasma renin, higher aldosterone, and higher aldosterone-to-renin ratio, indicative of renin-independent aldosteronism, experienced greater reductions in systolic BP with spironolactone.^12^ Similar findings were observed in 527 patients at risk for HF from the HOMAGE trial. Spironolactone 25–50 mg/day vs control reduced systolic BP by 10.3 mmHg over 9 months, and patients with lower renin had a greater BP reduction with spironolactone than those with higher renin (*P* for interaction = .041).^124^ Further explanatory analysis or trials consistently showed antihypertensive effects of spironolactone in patients with TRH who had underlying diseases such as primary aldosteronism, HF with preserved ejection fraction (HFpEF), and dialysis.^108–113^

**Table 2.** Blood pressure–lowering effects in aldosterone-targeted therapies

MRA	Study design	Population and inclusion criteria	Age	Female	T2D	eGFR	Study duration	Control/placebo group	*n*	MRA group	*n*	MRA vs control Mean ΔSBP (95%CI)	Year
**Steroidal MRAs**													
Spironolactone ^(11)^	Randomized, double-blind, placebo-controlled, crossover	TRH 335 patients with hypertension (office SBP ≥140 mmHg, home SBP ≥130 mmHg) despite maximally tolerated doses of three drugs (i.e. ACEi/ARB, CCB, and diuretic); eGFR <45mL/min/1.73 m^2^ were excluded	61	31	14	91	12 weeks	Placebo	274	Spironolactone 25–50 mg/day	285	−8.70 (−9.72 to −7.69)	2015
								Doxazosin	282	Spironolactone 25–50 mg/day	285	−4.03 (−5.04 to −3.02)	
								Bisoprolol	285	Spironolactone 25–50 mg/day	285	−4.48 (−5.50 to −3.46)	
Spironolactone ^(108)^	Post hoc analysis of randomized, double-blind, placebo-controlled	TRH and HFpEF 403 patients with HFpEF and hypertension as baseline SBP of 140–160 mmHg on ≥3 antihypertensive drugs; eGFR <30mL/min/1.73 m^2^ were excluded	72	61	52	63	32 weeks	Placebo	212	Spironolactone 25 mg/day	191	−6.1 (−8.9 to −3.3)	2018
Spironolactone ^(109)^	randomized, double-blind, placebo-controlled, parallel group	TRH 161 patients with hypertension (office BP ≥140/90 mmHg) despite three antihypertensive drugs (i.e. a diuretic); if office BP >130/80 mmHg, diabetes or CKD (serum creatinine >133 µmol/L or proteinuria >300 mg/day) were included.	60	38	38	Creatinine 81 µmol/L	8 weeks	Placebo	76	Spironolactone 25 mg/day	74	−10.50 (−14.63 to −6.37)	2014
Spironolactone ^(110)^	Randomized, double-blind, placebo-controlled, parallel group	TRH and dialysis 82 patients with hypertension (pre-dialysis BP >160/90 mmHg or post-dialysis BP >140/80 mmHg in haemodialysis patients and clinic BP ≥140/90 mm Hg in peritoneal patients) despite ≥3 antihypertensive drugs.	55	41	16	NA	12 weeks	Placebo	36	Spironolactone 25 mg/day	40	−17.0 (−18.0 to −16.0)	2014
Spironolactone ^(111)^	Randomized, double-blind, placebo-controlled, parallel group	TRH 119 patients with hypertension (office BP ≥130/80 mmHg) despite maximally tolerated doses of 3 drugs (i.e. a diuretic and an ACEi or ARB) and diabetes mellitus; eGFR <50 mL/min/1.73 m^2^ were excluded	64	22	100	79	16 weeks	Placebo	55	Spironolactone 25–50 mg/day	57	−8.9 (−13.2 to −4.7)	2013
Spironolactone ^(112)^	Randomized, placebo-controlled, parallel group	TRH and CKD 41 patients with hypertension despite ≥3 antihypertensive drugs (i.e. ACEi/ARB, CCB and diuretic) and eGFR 25–50 mL/min/1.73 m^2^	50	46	NA	Cr. 2.3 mg/dL	12 weeks	Placebo	19	Spironolactone 25–50 mg/day	22	−31.0 (−39.6 to −22.4)	2011
Spironolactone ^(113)^	Randomized, double-blind, crossover	Low renin hypertension 57 hypertensive patients with low renin (≤12mU/L) and elevated aldosterone–renin ratio (>750); hypertension (clinic BP 140–170/90–110 mmHg)	60	46	NA	NA	5 weeks	Amiloride 20–40 mg/day	51	Spironolactone 50–100 mg/day	51	0.08 (−2.97 to 3.11)	2007
								Bendroflumethiazide 2.5–5.0 mg/day	51	Spironolactone 50–100 mg/day	51	1.17 (−1.85 to 4.23)	
Eplerenone ^(114)^	Randomized, double-blind, placebo-controlled, parallel group	TRH 155 patients with hypertension despite ≥3 antihypertensive drugs (i.e. a diuretic); creatinine clearance <50 mL⁄min were excluded	58	38	23	81	8 weeks	Placebo	33	Eplerenone 50 mg/day (twice daily)	33	−9.90 (−13.76 to −6.04)	2013
Eplerenone ^(115)^	Randomized, double-blind, placebo-controlled, parallel group	TRH 51 patients with hypertension (office BP ≥140/90 mmHg) despite maximally tolerated doses of 3 drugs (i.e. a diuretic and an ACEi or ARB); diabetes mellitus and eGFR <50 mL/min/1.73 m^2^ were excluded	60	19	0	Creatinine 0.9 mg/dL	26 weeks	Placebo	26	Eplerenone 50 mg/day (twice daily)	25	−5.00 (−15.71 to 5.71)	2017
Eplerenone ^(116)^	Randomized, double-blind, placebo lead-in, parallel group	Essential hypertension 417 patients with hypertension (both seated, cuff-assessed DBP 95 mmHg and 114 mmHg and a 24-h mean DBP 85 mmHg); serum creatinine 1.5 mg/dL or serum K+ 5.0 mEq/L were excluded	NA	33	NA	NA	8 weeks	Placebo	53	Eplerenone 50 mg/day	54	−6.0	2002
								Placebo	53	Eplerenone 100 mg/day	49	−9.5	
								Placebo	53	Eplerenone 400 mg/day	56	−16.6	
								Placebo	53	Eplerenone 25 mg/day (twice daily)	55	−9.7	
								Placebo	53	Eplerenone 50 mg/day (twice daily)	54	−13.3	
								Placebo	53	Eplerenone 200 mg/day (twice daily)	48	−16.4	
								Spironolactone 50 mg/day (twice daily)	50	Eplerenone 200 mg/day (twice daily)	48	1.9	
Spironolactone/eplerenone ^(117)^	Randomized, double-blind, active-controlled, parallel group	Primary aldosteronism 141 patients with hypertension (seated DBP 90–120 mmHg and SBP <200 mmHg) and primary aldosteronism; serum creatinine >1.5 mg/dl in men and >1.3 mg/dl were excluded.	53	32	NA	NA	16 weeks	Eplerenone 100–300 mg/day	70	Spironolactone 75–225 mg/day	71	−17.1 (−23.4 to −10.8)	2011
**Non-steroidal MRAs**													
Finerenone ^(118)^	Randomized, double-blind, placebo-controlled, parallel group	Diabetic kidney disease 823 patients with Type 2 diabetes and CKD treated with an ACE-inhibitor or an ARB with at least the minimum recommended dose UACR >30 mg/g and an eGFR >30 mL/min/1.73 m^2^ serum K+ <4.8 mmol/L	64	22	100	68	90 days	Placebo	94	Finerenone 7.5 mg/day	97	−2.8 (−6.5 to 0.8)	2015
								Placebo	94	Finerenone 10 mg/day	98	0.1 (−3.5 to 3.8)	
								Placebo	94	Finerenone 15 mg/day	125	−5.1 (−8.5 to −1.7)	
								Placebo	94	Finerenone 20 mg/day	119	−4.7 (−8.2 to −1.3)	
Finerenone ^(55, 119)^	Randomized, double-blind, placebo-controlled, parallel group	Diabetic kidney disease 5734 patients with Type 2 diabetes and CKD treated with an ACE-inhibitor or an ARB at the maximum dose UACR 30–300 mg/g and an eGFR 25–60 mL/min/1.73 m^2^ UACR 300–5000 mg/g and an eGFR 25–75 mL/min/1.73 m^2^ serum K+ <4.8 mmol/L	66	30	100	44	48 weeks	Placebo	2841	Finerenone 10–20 mg/day	2833	−2.7 (−3.3 to −2.1)	2020
Finerenone ^(56)^	Randomized, double-blind, placebo-controlled, parallel group	Diabetic kidney disease 7437 patients with Type 2 diabetes and CKD treated with an ACE-inhibitor or an ARB at the maximum dose UACR 30–300 mg/g and an eGFR 25–90 mL/min/1.73 m^2^ UACR 300–5000 mg/g and an eGFR >60 mL/min/1.73 m^2^ serum K+ <4.8 mmol/L	64	31	100	68	48 weeks	Placebo	3666	Finerenone 10–20 mg/day	3686	−2.71 (−3.16 to −2.27)	2021
Finerenone ^(120)^	Post hoc analysis of randomized, double-blind, placebo-controlled	Diabetic kidney disease and TRH 624 patients with hypertension (office SBP 135–160 mmHg) despite maximally tolerated doses of three drugs (i.e. ACEi/ARB and diuretic) and eGFR of 25–45 mL/min/1.73 m^2^	68	35	100	36	17 weeks	Placebo	308	Finerenone 10–20 mg/day	316	−5.74 (−7.99 to −3.49)	2023
Ocedurenone (126)	Randomized, double-blind, placebo-controlled, parallel group	TRH 162 patients with hypertension (clinic SBP 140–179 mmHg) despite maximally tolerated doses of two drugs (i.e. diuretic) eGFR 15–45 mL/min/1.73 m^2^	65	45	57	32	84 days	Placebo	57	Ocedurenone 0.25 mg/day	51	−7.0 (3.37)	2021
								Placebo	57	Ocedurenone 0.5 mg/day	54	−10.2 (3.32)	
Esaxerenone ^(121)^	Randomized, double-blind, parallel group	Essential hypertension 1001 patients with hypertension (clinic BP 140–179/90–109 mmHg during the washout period of antihypertensive agents, and ambulatory BP ≥130/80 mmHg at Week 3 of the washout period) diabetes with albuminuria, serum K+ <3.5 mEq/L or ≥5.1 mEq/L, or eGFR <60 mL/min/1.73 m^2^ were excluded	56	28	16	79	12 weeks	Eplerenone 50 mg/day	331	Esaxerenone 2.5 mg/day	330	−1.6 (−3.3 to 0.1)	2020
								Eplerenone 50 mg/day	331	Esaxerenone 5.0 mg/day	337	−4.8 (−6.4 to −3.1)	
**Aldosterone Synthase Inhibition**													
Baxdrostat ^(122)^	Randomized, double-blind, parallel group	TRH 275 patients with hypertension (clinic BP >130/80 mmHg) despite maximally tolerated doses of three antihypertensive drugs (i.e. diuretic). A seated BP >180/110 mmHg, eGFR <45 mL/min/1.73 m^2^, or uncontrolled diabetes were excluded.	64	59	41	86	12 weeks	Placebo	69	Baxdrostat 0.5 mg/day	69	**-**	2022
								Placebo	69	Baxdrostat 1.0 mg/day	70	−8.1 (−13.5 to −2.8)	
								Placebo	69	Baxdrostat 2.0 mg/day	67	−11.0 (−16.4 to −5.5)	
Lorundrostat ^(123)^	Randomized, double-blind, parallel group	TRH 200 patients with hypertension (clinic SBP> 130 mmHg) despite maximally tolerated doses of two drugs were divided into the following two categories.					8 weeks	Placebo	29	Lorundrostat 12.5 mg/day	19	−1.5 (−8.3 to 5.3)	2023
		163 patients who had suppressed plasma renin activity (<1.0 ng/mL/h) and elevated plasma aldosterone (>1.0 ng/dL).	63	57	47	82		Placebo	29	Lorundrostat 12.5 mg/day (twice daily)	19	−7.2 (−14.0 to −0.4)	
								Placebo	29	Lorundrostat 25 mg/day (twice daily)	28	−7.0 (−13.1 to −0.8)	
								Placebo	29	Lorundrostat 50 mg/day	28	−9.6 (−15.8 to −3.4)	
								Placebo	29	Lorundrostat 100 mg/day	25	−7.8 (−14.1 to −1.5)	
		37 patients without suppressed plasma renin activity (>1.0 ng/mL/h) as an explanatory analysis	63	67	33	84		Placebo	6	Lorundrostat 100 mg/day	31	−11.4 (2.5) mmHg decrease in SBP in the lorundrostat group	

Age and eGFR (or serum creatinine) are reported as mean value.

ACEi, angiotensin-converting enzyme inhibitor; ARB, angiotensin receptor blocker; CCB, calcium-channel blocker; CI, confidence interval; CKD, chronic kidney disease; DBP, diastolic blood pressure; eGFR, estimated glomerular filtration rate; HFpEF, heart failure with preserved ejection fraction; MRA, mineralocorticoid receptor antagonist; NA, not available; SBP, systolic blood pressure; T2D, Type II diabetes; TRH, treatment-resistant hypertension; UACR, urine albumin-to-creatinine ratio.

Amiloride, an ENaC inhibitor, reduced home systolic BP similarly to spironolactone (20.4 mmHg vs 18.3 mmHg) in 46.5% (*n* = 146) of patients with TRH from a PATHWAY-2 substudy.^12^ Although the open-label assessment conducted after the double-blind trial and the small sample size limited the conclusions, amiloride is suggested as an alternative for patients with TRH who cannot tolerate spironolactone.^125^

### Eplerenone

Several trials suggest that the antihypertensive effects of eplerenone may be modest.^115–117^ In a study of 417 patients with essential hypertension, spironolactone 100 mg/day showed a more pronounced BP-lowering effect compared with eplerenone 400 mg/day over 8 weeks (−16.7 vs −14.8 mmHg).^116^ In another study with 141 patients with primary aldosteronism, spironolactone 75–225 mg/day significantly decreased clinic systolic BP by 17.1 mmHg, compared with eplerenone 100–300 mg/day over 16 weeks.^117^ However, there is also clear evidence of a significant antihypertensive effect of eplerenone.^114,126,127^ Eplerenone 200 mg/day significantly reduced BP and further regressed left ventricular mass, comparable to enalapril 40 mg/day over 9 months. Notably, the combination of eplerenone and enalapril pronounced their antihypertensive and anti-remodelling effects.^126^ Additionally, among 52 patients with TRH, eplerenone at average 70 mg/day significantly decreased clinic systolic BP by 17.6 mmHg and 24-h mean systolic BP by 12.2 mmHg over 12 weeks.^127^ In a meta-analysis of 5 trials including 1437 patients with hypertension (8–16 weeks), eplerenone 50–200 mg/day vs placebo decreased systolic BP by 9.21 mmHg, with no significant difference across these doses, while a dose of 25 mg/day showed no significant BP reduction.^128^

### Finerenone

Finerenone 10–20 mg/day vs placebo had modest BP-lowering effects of 3–4 mmHg in patients with Type 2 diabetes (T2D) and CKD, as consistently reported in the ARTS-DN, FIDELIO-DKD, and FIGARO-DKD trials.^55,56,118,119^ In the ARTS-DN trial, from baseline to Day 90, finerenone did not show meaningful dose-dependent effects on lowering BP overall.^118^ However, in a sub-analysis of this trial, finerenone vs placebo persistently reduced BP across the entire 24-h period, despite its once-daily dosing regimen and relatively short half-life.^129^

### Ocedurenone

Ocedurenone appeared to be a promising antihypertensive agent for TRH and CKD. In the BLOCK-CKD trial including 162 patients with uncontrolled hypertension and Stage 3b/4 CKD, ocedurenone vs placebo significantly reduced systolic BP over 84 days in a dose-dependent manner and did not significantly increase the occurrence of hyperkalaemia.^130,131^ However, intriguingly, a pre-specified interim analysis of a larger trial, CLARION-CKD trial showed a failure to achieve significant changes in systolic BP from baseline to week 12, leading to the trial's termination.^132^ Therefore, unless one figures out the discrepancy of results between BLOCK-CKD and CLARION-CKD trials, future developments of ocedurenone may be at stake.

### Esaxerenone

Esaxerenone, a non-steroidal MRA, was approved for essential hypertension only in Japan. In the ESAX-HTN trial including 1101 patients with essential hypertension, esaxerenone decreased BP in a dose-dependent manner, and the magnitude of lowering BP with esaxerenone 5 mg/day was greater than that with eplerenone 50 mg/day (systolic BP −4.8 mmHg, *P* < .0001).^121^

## Reno-protective effects of mineralocorticoid receptor antagonists in clinical trials

### Steroidal mineralocorticoid receptor antagonists

A meta-analysis of 14 studies, which included 1193 adults with eGFR >15 mL/min/1.73 m^2^ and proteinuria on ACEi/ARB, showed that spironolactone or eplerenone reduced proteinuria compared with placebo or standard care over a median of 3.5 months.^139^ In 130 patients with T2D and CKD (eGFR ≥30 mL/min/1.73 m^2^ and UACR ≥30 mg/g), spironolactone 12.5 mg/day vs control decreased UACR levels over 24 weeks.^140^ In 140 patients with T2D at high risk of or with CV diseases, eplerenone 100–200 mg/day vs placebo decreased UACR levels by 34% over 26 weeks.^141^ Similarly, in 1175 patients with HFpEF from the TOPCAT trial, after correcting for the placebo response, spironolactone reduced UACR levels by 39% at the 1-year visit compared with baseline in the overall population, and by 76% in those with severely increased albuminuria levels (UACR >300 mg/day).^142^ In post hoc analyses of the EMPHASIS-HF and TOPCAT Americas trials, MRAs caused a decrease in eGFR within weeks of treatment initiation but did not alter the long-term trajectories of eGFR in both HFrEF and HFpEF populations (*Table 3*).^134^ Similar eGFR trajectories with MRAs were observed in patients with left ventricular ejection fraction (LVEF) < 40% after myocardial infarction (MI).^133^ Importantly, the acute eGFR decline is haemodynamically mediated and neither injures kidney itself nor negates clinical benefits of MRAs, as evidenced by clinical trials.^133,143–146^

**Table 3.** Effects of mineralocorticoid receptor antagonists on glomerular filtration rate slope and renal composite outcome

Trial, year	Participants at baseline	Participants at last follow-up, *n* (%)	Median follow-up period, months	Baseline eGFR, mL/min/1.73 m^2^	Mean doses of study drug (mg)	GFR slope (period/Δ eGFR)				Renal outcomes
						Acute phase		Chronic phase		HR (95%CI) for TTx
						MRA	placebo	MRA	placebo	
**EPHESUS, 2003** ^(57, 133)^	5792 patients with a LVEF≤40% after acute MI who had signs and symptoms of HF or diabetes; eGFR ≥30 min/min/1.73 m^2^ and serum potassium ≤5.0 mmol/L	378 (6.5)	24	70 ± 21	42.6	Baseline to Month 1		Month 1 to end of study		NA
						Eplerenone	Placebo	Eplerenone	Placebo	
						NA	NA	mean ΔeGFR = −2.7	mean ΔeGFR = −1.8	
						Difference = −1.3 ± 0.4		mean ΔeGFR = −0.9		
**EMPHASIS-HF, 2011** ^(59, 134)^	2713 patients with HF and an LVEF <30% (or, if >30 to 35%, a QRS duration of >130 msec on electrocardiography), NYHA II; eGFR ≥30 min/min/1.73 m^2^ and serum potassium ≤5.0 mmol/L	1122 (41.4)	22	66 ± 18	39.1	Baseline to Months 4–6		Months 4–6 to end of study		
						Eplerenone	Placebo	Eplerenone	Placebo	
						−2.5 (−3.2 to −1.8)	0.1 (−0.7 to 0.8)	−0.8 (−1.5 to −0.1)	−0.6 (−1.4 to 0.2)	
						difference = −2.4 (−3.4 to −1.4)		difference = −0.3 (−1.3 to 0.7)		
**TOPCAT (Americas Region), 2014** ^(134–136)^	1739 patients with symptomatic HF and an LVEF≥45%; eGFR ≥30 min/min/1.73 m^2^, and serum potassium ≤5.0 mmol/L	426 (24.5)	40	58 ± 19	21.7	Baseline to Month 4–6		Month 4–6 to End of study		
						Spironolactone	Placebo	Spironolactone	Placebo	
						−1.8 (−2.6 to −0.8)	0.1 (−0.8 to 0.9)	−0.4 (−1.5 to 0.6)	−0.6 (−1.7 to 0.5)	
						difference = −2.0 (−3.0 to −1.8)		difference = 0.1 (−1.4 to 1.7)		
**FIDELIO-DKD, 2020** ^(55)^	5599 patients with T2D and CKD treated with an ACE-inhibitor or an ARB at the maximum dose; UACR 30–300 mg/g and an eGFR 25–60 mL/min/1.73 m^2^ or UACR 300–5000 mg/g and an eGFR 25–75 mL/min/1.73 m^2^; and serum K+ <4.8 mmol/L	675 (12.0)	44	44 ± 13	15.1	Baseline to Month 4		Month 4 to End of study		Kidney failure^a^, >40% decline in eGFR, and renal-cause mortality (primary outcome)
						Finerenone	Placebo	Finerenone	Placebo	
						−3.2 (−3.4 to −2.9)	−0.7 (−1.0 to −0.4)	−2.7 (−3.0 to −2.4)	−4.0 (−4.3 to −3.7)	
						difference = −2.5		difference = 1.3		HR = 0.82 (0.73–0.93)
**FIGARO-DKD, 2021** ^(56, 137)^	7351 patients with T2D and CKD treated with an ACE-inhibitor or an ARB at the maximum dose; UACR 30–300 mg/g and an eGFR 25–90 mL/min/1.73 m^2^ or UACR 300–5000 mg/g and an eGFR>60 mL/min/1.73 m^2^; and serum K+ <4.8 mmol/L	1658 (22.6)	48	68 ± 22	17.5	Baseline to Month 4		Month 4 to End of study		Kidney failure^a^, >40% decline in eGFR and renal-cause mortality (secondary outcome)
						Finerenone	Placebo	Finerenone	Placebo	
						−3.5 (−3.8 to −3.1)	−1.2 (−1.5 to −0.9)	−2.4 (−2.8 to −2.0)	−3.5 (−3.9 to −3.1)	
						Difference = −2.2 (−2.7 to −1.8)		Difference = 1.1 (0.6 to 1.7)		HR = 0.87 (0.76–1.01)
**FINEARTS-HF, 2024** ^(60, 138)^	6001 patients with symptomatic HF and an LVEF≥40%; eGFR ≥25 min/min/1.73 m^2^ and serum potassium ≤5.0 mmol/L	NA	32	62 ± 20	NA	Baseline to Month 3		Month 3 to end of study		Kidney failure^a^, >50% decline in eGFR, and sustained decline in eGFR to <15mL/min/1.73 m^2^
						Finerenone	Placebo	Finerenone	Placebo	
						−3.0 (−3.4 to −2.7)	−0.1 (−0.5 to 0.2)	−0.9 (−1.1 to −0.7)	−1.1 (−1.3 to −0.9)	
						Difference = −2.9 (−3.4 to −2.4)		Difference = 0.2 (−0.1 to 0.4)		HR = 1.33 (0.94–1.89)

^a^Kidney failure; eGFR <15 mL/min/1.73 m^2^, long-term dialysis, or kidney transplantation

ACEi, angiotensin-converting enzyme inhibitor; ARB, angiotensin receptor blocker; CCB, calcium-channel blocker; CI, confidence interval; CKD, chronic kidney disease; eGFR, estimated glomerular filtration rate; HF, heart failure; HR, hazard ratio; K+, potassium; LVEF, left ventricular ejection fraction; MI, myocardial infarction; MRA, mineralocorticoid receptor antagonist; NA, not available; NYHA, New York Heart Association; T2D, Type II diabetes; TTx, treatment; UACR, urine albumin-to-creatinine ratio.

The efficacy and safety of spironolactone in chronic haemodialysis patients remain under investigation.^147–149^ In the ALCHEMIST trial (*n* = 644, NCT01848639), spironolactone did not reduce primary CV outcomes but lowered the risk of HF hospitalization compared with placebo and was well-tolerated (Rossignol, unpublished data). The ongoing larger Phase III trial, ACHIEVE (NCT03020303), aims to further evaluate the efficacy and safety of spironolactone in dialysis patients, with the goal of providing more clarity in this population.

### Non-steroidal mineralocorticoid receptor antagonists

Overall, finerenone has shown reno-protective benefits particularly in patients with T2D and CKD. In Phase 2 trial of the ARTS-DN trial, among 823 patients with T2D and CKD, finerenone 7.5–20 mg/day vs placebo reduced UACR in a dose-dependent manner.^118^ Subsequently, in two trials for CKD and T2D (FIDELIO-DKD and FIGARO-DKD), finerenone vs placebo reduced a risk of composite kidney outcome and slowed a rate of eGFR decline over the follow-up period.^55,56,137^ Furthermore, among 13 026 patients in the FIDELITY analysis, a meta-analysis of FIDELIO-DKD and FIGARO-DKD, finerenone vs placebo reduced the risk of ESKD by 20%, regardless of baseline renal function (i.e. eGFR and/or UACR categories).^150^ In the FINEARTS-HF trial, finerenone vs placebo did not impact the composite kidney outcome (i.e. ESKD, sustained 50% decrease in eGFR, or sustained eGFR decline to <15 mL/min/1.73 m^2^) but reduced albuminuria and the risk of new-onset micro- or macroalbuminuria in patients with HF and LVEF ≥40%.^138^

In adults with CKD (UACR 200–3500 mg/g and eGFR 25–90 mL/min/1.73 m^2^) without T2D, the ongoing Phase II FIND-CKD trial (*n* = 1584) is evaluating the effect of finerenone vs placebo on the mean annual rate of change in eGFR over 32 months.^151^

Esaxerenone may also have reno-protective effects. In a Japanese trial that included 455 patients with T2D and CKD, the treatment with esaxerenone reduced the UACR levels compared with placebo over 52 weeks (geometric least-squares mean ratio = 0.38, 95% CI 0.33–0.44).^152^

## Potential cardiovascular benefits from mineralocorticoid receptor antagonists in chronic kidney disease subgroups

### Steroidal mineralocorticoid receptor antagonists

Spironolactone or eplerenone provide robust and consistent CV benefits in patients with HFrEF, those with LVEF <40% after MI or those with acute MI,^57–59,153,154^ and may have the potential for CV risk reduction in those with HFpEF (^135,136^) Across clinical trials of MRA therapy, the prevalence of CKD varied. However, overall, CV benefits from spironolactone and eplerenone remained consistent regardless of CKD status or severity (*Table 4*). However, it must be emphasized that in most steroidal MRAs trials, patients with low eGFR were excluded (*Table 4*). In a sub-analysis of the EMPHASIS-HF trial, when eplerenone doses were stratified by the renal function severity, its effects on CV outcomes were similar between patients with impaired and preserved renal function.^155^ Additionally, in a meta-analysis of the RALES, EMPHASIS-HF, TOPCAT Americas, and the EPHESUS trials, MRAs vs placebo reduced the composite of CV death or HF hospitalization across a wide spectrum of eGFR; however, the effect of MRAs was attenuated as eGFR decreased, leading to neutralization in those with an eGFR ≤30 mL/min/1.73 m^2^ (*P* for trend = .033).^156^

**Table 4.** Effects of mineralocorticoid receptor antagonists on primary outcome across kidney function categories

Trials, year	Population	Drug (vs placebo)	Kidney function exclusion criteria (lowest eGFR value)	Baseline eGFR, mL/min/1.73 m^2^	Baseline eGFR<60 mL/min, %	Baseline UACR, mg/g	Baseline LVEF, %	Follow-up period, months	Primary outcome HR (95%CI) for TTx	Kidney function strata	Hazard ratio	*P* for interaction
**RALES, 1999** ^(58)^	1663 patients with HFrEF and severe symptom	Spironolactone, −25 mg/day	Creatinine >2.5 mg/dL	65 ± 23	48	NA	25 ± 7	24	All-cause death	eGFR ≥60	0.71 (0.57–0.90)	NA
									HR = 0.70 (0.60–0.82)	eGFR <60	0.68 (0.56–0.84)	
**EPHESUS, 2003** ^(57)^	6642 patients with HFrEF after MI	Eplerenone, −50 mg/day	Creatinine >2.5 mg/dL	70 ± 21	33	NA	33 ± 6	16	CV death, non-fatal MI, stroke, and HF hospitalization	Cr<1.1 mg/dl	NA	0.53
									HR = 0.87 (0.79–0.95)	Cr≥1.1 mg/dl	NA	
**EMPHASIS-HF, 2011** ^(59)^	2737 patients with HFrEF and mild symptom	Eplerenone, −50 mg/day	eGFR <30 mL/min/1.73 m^2^	70 ± 22	35	NA	26 ± 5	21	CV death or HF hospitalization	eGFR ≥60	NA	0.50
									HR = 0.63 (0.54–0.74)	eGFR <60	NA	
**TOPCAT Americas, 2014** ^(135, 136)^	1767 patients with symptomatic HFpEF	Spironolactone, 15–45 mg/day	eGFR<30 mL/min/1.73 m^2^ or creatinine ≥2.5 mg/dL	64 ± 22	53	27 (9–117)	58 ± 8	40	CV death, HF hospitalization, or aborted cardiac arrest	eGFR >60	0.66 (0.50–0.88)	0.13
										eGFR 45–60	0.99 (0.73–1.36)	
									HR = 0.82 (0.69–0.98)	eGFR <45	0.89 (0.66–1.21)	
**REMINDER, 2014** ^(153)^	1012 patients with STEMI	Eplerenone, 25–50 mg/day	eGFR ≤30 mL/min/1.73 m^2^	86 ± 25	7.8	NA	NA	11	CV-related outcome^a^	eGFR ≥60	NA	0.47
									HR = 0.58 (0.45–0.75)	eGFR <60	NA	
**ALBATROSS, 2016** ^(154)^	1603 patients with STEMI or NSTEMI	MRA regimen (a single 200 mg potassium canrenoate IV bolus plus spironolactone 25 mg/day for 6 months)	Creatinine >220 µmol/L	Ccr, 101 (77–121) mL/min	NA	NA	50 (45–60)	6	All-cause death, VT/VF, ICD indication, or HF hospitalization	Ccr >60 mil/min	0.92 (0.67–1.27)	0.64
									HR = 0.97 (0.73–1.28)	Ccr ≤60 mil/min	1.08 (0.59–2.00)	
**FIDELIO-DKD, 2020** ^(55)^	5734 patients with CKD and T2D	Finerenone, 10–20 mg/day	eGFR <25 mL/min/1.73 m^2^	44 ± 13	88	852 (446–1634)	NA	31	kidney failure (i.e. eGFR <15 mL/min, long-term dialysis or kidney transplantation), >40% decline in eGFR, and renal-cause mortality	eGFR ≥60	0.78 (0.55–1.12)	NA
										eGFR 45–60	0.77 (0.61–0.96)	
										eGFR 25–45	0.86 (0.73–1.00)	
									HR = 0.82 (0.73–0.93)	eGFR <25	0.88 (0.48–1.64)	
**FIGARO-DKD, 2021** ^(56)^	7437 patients with CKD and T2D	Finerenone, 10–20 mg/day	eGFR <25 mL/min/1.73 m^2^	68 ± 22	38	308 (108–740)	NA	41	CV death or non-fatal MI, stroke, or HF hospitalization	eGFR ≥60	0.87 (0.73–1.03)	NA
										eGFR 45–60	0.81 (0.61–1.06)	
									HR = 0.87 (0.76–0.98)	eGFR 25–45	0.95 (0.73–1.25)	
										eGFR <25	0.62 (0.10–3.83)	
**FINEARTS-HF, 2024** ^(60)^	6001 patients with symptomatic HFpEF	Finerenone, 20–40 mg/day	eGFR <25 mL/min/1.73 m^2^	62 ± 20	48	18 (7–67)	53 ± 8	32	CV death or total worsening HF events (i.e. unplanned hospitalization or urgent visit)	eGFR ≥60	0.72 (0.59–0.88)	NA
									HR = 0.84 (0.74–0.95)	eGFR <60	0.91 (0.78–1.07)	

eGFR and LVEF are reported as mean ± SD and UACR as median (25th–75th percentile). In ALBATROSS, eGFR and LVEF are reported as median (25th–75th percentile). In RALES, EPHESUS, and ALBATROSS, the exclusion criteria for kidney function are defined by serum creatinine concentrations.

^a^CV outcome: CV death, HF hospitalization, VT/VF, LVEF ≤40%, or BNP >200 pg/mL or NT-proBNP >450 pg/mL (in patients aged <50 years), >900 pg/mL (in patients aged 50–75 years), or 1800 pg/mL (patients aged >75 years) after 1 month.

BNP, b-type natriuretic peptide; Ccr, creatinine clearance; CKD, chronic kidney disease; CV, cardiovascular; eGFR, estimated glomerular filtration rate; HFpEF, heart failure with preserved ejection fraction; HFrEF, heart failure with reduced ejection fraction; HR, hazard ratio; ICD, implantable cardioverter defibrillator; LVEF, left ventricular ejection fraction; MI, myocardial infarction; MRA, mineralocorticoid receptor antagonist; NA, not available; NSTEMI, non-ST-elevation MI; NT-proBNP, N-terminal pro-b-type natriuretic peptide; STEMI, ST-elevation MI; T2D, Type 2 diabetes; UACR, urine albumin-to-creatinine ratio; VF, ventricular fibrillation; VT, ventricular tachycardia.

Despite declines in kidney function after MRA initiation, the cardioprotective effects of steroidal MRAs generally appear to be maintained. In a sub-analysis of the RALES trial, patients facing worsening renal function (WRF, > 30% decline in eGFR) by week 12 had an increased risk of all-cause mortality with placebo, whereas the risk was mitigated in those receiving spironolactone (*P* for interaction = .009).^143^ Similar findings were also reported in sub-analyses of the EPHESUS, EMPHASIS-HF, and TOPCAT trials, highlighting the persistent cardioprotective effects of MRA therapy despite occurrences of WRF.^133,144,145^ Furthermore, in a meta-analysis of the RALES and EMPHASIS-HF trials (*n* = 4355), 6.8% of patients experienced eGFR deterioration to <30 mL/min/1.73 m^2^ after randomization. These patients showed a similar relative reduction in CV risk with MRAs compared with those who maintained eGFR ≥30 mL/min/1.73 m^2^ throughout the study.^157^

### Finerenone

Finerenone has shown CV benefits in patients with T2D and over a wide range of CKD in the FIGARO-DKD and FIDELIO-DKD trials, and its effects on the primary outcomes were consistent regardless of baseline renal function (*Table 4*).^55,56^ In the FIGARO-DKD trial and FIDELITY programme focused on the effect of finerenone on HF outcomes, highlighting that finerenone vs placebo consistently reduced risks of HF outcomes (i.e. first, or recurrent HF hospitalizations).^158,159^ In FIDELITY (*n* = 13 023), 6.8% had a baseline eGFR <30 mL/min/1.73 m^2^; however, the relative risk reduction of CV outcomes with finerenone vs placebo was similar for both eGFR groups (*P* for interaction = .67).^160^

In the FINEARTS-HF trial, finerenone vs placebo reduced the risk of composite of total worsening HF events (a first or recurrent unplanned hospitalization or urgent visit for HF) and CV death by 16% in patients with HFpEF.^60^ The baseline eGFR levels were comparable to those reported in other HFpEF trials,^161^ 13.6% were on SGLT2is, and CV benefit from finerenone was consistent regardless of baseline eGFR categories (eGFR <60 and ≥60 mL/min/1.73 m^2^).^60^

## Hyperkalaemia or renal failure associated with mineralocorticoid receptor antagonist in chronic kidney disease

Echoing the results of the PATHWAY-2 trial, patients with TRH and an eGFR <45 mL/min/1.73 m^2^ are less likely to receive MRA therapy due to concerns about hyperkalaemia-related rhythm disturbance,^162,163^ and the concerns has been overly magnified.^164^ It should be noticeable that MRA discontinuation after hyperkalaemia reduced its recurrence but significantly increased subsequent CV risk.^165^ While potassium binders mitigate a risk of hyperkalaemia, long-term data on tolerability and adherence remain unverified in randomized trials except for patiromer.^166^ In the AMBER trial including 295 patients with TRH and eGFR 25–45 mL/min/1.73 m^2^, those with the concomitant use of patiromer vs placebo remained on spironolactone at week 12 (86% in the patiromer group vs 66% in the control group; between-group difference = 19.5%, 95% CI 10.0–29.0; *P* < .0001).^167^ Some data suggested a lower rate of hyperkalaemia with finerenone vs steroidal MRA, but head-to-head comparison data are limited.^120,168^ In the ARTS trial, finerenone resulted in less hyperkalaemia than spironolactone, although the spironolactone was forced titration per protocol to the high dose of 50 mg/day which is superior to the routine average dose of 25 mg.^169^ In the ARTS-HF trial, finerenone caused a smaller increase in serum potassium levels than eplerenone, although the rate of hyperkalaemia was similar between the two agents.^170^ Importantly, both trials limited finerenone doses to ≤20 mg/day and had relatively short follow-up periods. In the FINEARTS-HF trial, when finerenone 40 mg/day was administered to patients with an eGFR >60 mL/min/1.73 m^2^, the excess risk of hyperkalaemia compared with placebo was similar to what was reported in other steroidal MRA trials.^171^ Because cross trial indirect comparisons are challenging, further studies, including real world head-to-head comparisons, are needed to better compare the impact of steroid or non-steroid MRAs on hyperkalaemia.

### Thiazide, thiazide-like diuretics, and loop diuretics

Chlorthalidone, a thiazide-like diuretic, is often used to lower BP and may help mitigate the risk of hyperkalaemia in patients with CKD.^172,173^ In the CLICK trial including 160 patients with uncontrolled hypertension and Stage 4 CKD, the addition of chlorthalidone 12.5 mg/day vs placebo lowered 24-h ambulatory systolic BP by 10–14 mmHg and decreased albuminuria levels over 12 weeks. However, chlorthalidone also significantly increased plasma renin and aldosterone levels,^174^ and co-administration of MRAs has been suggested.^175^ Switching from hydrochlorothiazide, a thiazide, to chlorthalidone resulted in additional BP reduction in several studies.^176,177^ However, in a trial of 13 523 hypertensive patients on hydrochlorothiazide, the rates of primary CV outcomes and acute kidney injury-related hospitalizations were similar between those who continued hydrochlorothiazide and those switched to chlorothiazide.^178^

Furthermore, in patients with GFR <30 mL/min/1.73 m^2^, an adequately up-titrated loop diuretic is an option for managing TRH.^2,179^ Torsemide, a long-acting loop diuretic, inhibited aldosterone secretion by adrenal cells in a preclinical study^180^ and may be preferable to shorter-acting agents such as bumetanide or furosemide.^2^

### Sodium–glucose co-transporter 2 inhibitor

Sodium–glucose co-transporter 2 inhibitor has a modest and dose-independent BP-lowering effect in patients with TRH and CKD,^181^ although SGLT2i reduced risk of CV death in CKD.^182^ A combination of SGLT2is may reduce the risk of hyperkalaemia-associated outcomes in MRA users with concomitant CKD. In the FIDELITY analysis, patients on SGLT2is had numerically lower rates of serious hyperkalaemia (potassium ≥ 6.0 mmol/L) after initiating finerenone, compared with those not.^183^ In a meta-analysis of 6 trials, which included 49 875 patients with T2D at high CV risk or with CKD, SGLT2is reduced the risk of serious hyperkalaemia (potassium ≥6.0 mmol/L), compared with placebo across studies (HR 0.84, 95% CI 0.76–0.93; *P* for heterogeneity = .71). The risk reduction of hyperkalaemia with SGLT2is was consistent regardless of MRA use (*P* for heterogeneity >0.10).^184^ In a randomized crossover trial in patients with CKD (UACR ≥100 mg/g and eGFR 30–90 mL/min/1.73 m^2^), a combination of dapagliflozin 10 mg/day and eplerenone 50 mg/day significantly reduced risk of hyperkalaemia, compared with eplerenone 50 mg/day only during 4-week treatment periods.^185^

Balcinrenone, a selective MR modulator, was studied alongside dapagliflozin to assess their combined effects on UACR changes compared with dapagliflozin alone over 12 weeks in patients with symptomatic HF and LVEF <60% and CKD. Their findings were inclusive due to slow recruitment and a limited sample size.^186^ In a sub-analysis of the FINEARTS-HF trial, a similar risk of hyperkalaemia with finerenone was observed, even in patients on SGLT2is. However, these results should be interpreted cautiously due to potential indication bias among SGLT2i users with a high risk of hyperkalaemia.^187^ The ongoing Phase 2 trial, CONFIDENCE (NCT05254002) may provide the efficacy and safety of dual therapy with finerenone and SGLT2i in patients with T2D and CKD.^188^

## Aldosterone synthase inhibition

Mineralocorticoid receptor antagonists were associated with a reactive increase in aldosterone which may have MR-independent effects and exert much rapid effects by an alternative receptor.^189,190^ Aldosterone synthase inhibitor can limit aldosterone-related adverse effects by suppressing an hormone synthesis rather than blocking the MR^191^ (*Figure 1*).

In the BrigHTN trial including 248 patients with TRH and an eGFR ≥45 mL/min/1.73 m^2^, baxdrostat vs placebo reduced systolic BP dose-dependently over 12 weeks and decreased serum and urine aldosterone levels, without affecting serum cortisol. In two patients from the baxdrostat group, serum potassium ≥6.0 mmol/L was observed but did not recur after the drug withdrawal and reinitiation.^122^ However, in the HALO trial including 249 patients with uncontrolled hypertension, baxdrostat (0.5–2 mg/day) did not significantly reduce BP compared with placebo. A large placebo effect and low adherence to the study drug at some sites may impact its efficacy (Bhatt, unpublished data). Lorundrostat, another ASI agent, was studied in the Target-HTN trial, which included 200 patients with uncontrolled hypertension. Their results showed that lorundrostat vs placebo dose-dependently and consistently reduced systolic BP over 8 weeks regardless of baseline plasma renin activity. Severe hyperkalaemia (potassium ≥ 6.0 mmol/L) was uncommon.^123^ Blood pressure–lowering effects of ASI are summarized in *Table 2*.

Aldosterone synthase inhibitor may also have reno-protective effect. In a Phase 2 trial including 586 people with CKD (eGFR 30–90 mL/min/1.73 m^2^ and UACR 200–5000 mg/g) on an ACEi/ARB, BI690517 significantly and dose-dependently reduced UACR levels compared with placebo despite concurrent empagliflozin use.^192^ Further comparative studies will be needed to determine whether ASIs are equivalent or superior to MRAs in terms of clinical outcomes. Additionally, despite the distinct molecular and hormonal mechanisms between ASIs and MRAs targeting the aldosterone pathway,^193^ monitoring serum potassium and creatinine after the initiation of ASIs will remain necessary, as is the case with MRA therapy.

## Potential mechanisms behind the benefits of aldosterone-targeted therapy

The benefits of MRAs stem from its ability to block aldosterone and cortisol on the MRs, reducing inflammatory cytokines, tissue fibrosis, vascular and ventricular remodelling, and glomerular hypertrophy.^30,74^ These mechanisms are supported by studies using extracellular matrix turnover markers such as procollagen Type III amino-terminal peptide (PIIINP) and procollagen Type I carboxy-terminal peptide (PICP), which correlate with the severity of myocardial and renal interstitial fibrosis in biopsy samples.^194–196^

In a subset of the RALES trial (*n* = 261), serum PIIINP significantly decreased at 6 months in patients on spironolactone but not placebo, and the effect of spironolactone on all-cause mortality was pronounced among patients with above-median baseline levels of the markers.^197^ Similarly, in a subset of the EPHESUS trial, only patients receiving eplerenone had significantly lower serum PIIINP at 6 months (*n* = 476) and serum PICP at 9 months (*n* = 227) than baseline.^198,199^ Additionally, in the HOMAGE trial, which included 527 patients at risk of developing HF, spironolactone vs control significantly reduced serum PICP over 9 months,^200^ and the decline in serum PICP with spironolactone was correlated with improved diastolic dysfunction.^201^ Furthermore, in a meta-analysis of the HOMAGE, TOPCAT, and ALDO-DHF trials, which included 1038 patients with HFpEF with different severity, spironolactone vs placebo/usual care reduced serum PICP over 9–12 months.^202^

There are no supporting data regarding anti-fibrotic effects of ASIs. However, 11β-HSD2 is absent particularly in cardiomyocytes and podocytes, and cortisol, which is far more abundant than aldosterone, is essential for blocking the MRs.^47,203^ Therefore, different approaches from ASIs and MRAs were supported depending on the underlying diseases. If aldosterone is elevated (e.g. primary aldosteronism), an ASI is preferred. If MR activation dominates (e.g. HF), an MRA is favoured. When both are elevated (e.g. TRH and CKD), both agents may be needed.^204^ However, these assumptions need to be investigated. Therapies with ASI are being investigated in dedicated trials of HF and CKD by combining them with SGLT2is (empagliflozin or dapagliflozin) (EASi-HF-Preserved, NCT06424288; EASi-KIDNEY, NCT06531824; and NCT06268873).

## Future trial design in treatment-resistant hypertension and chronic kidney disease

Established evidence shows the synergically high risk of CV and renal events in patients with TRH and CKD, significantly mediated by aldosterone and activation of MRs. Whether aldosterone-targeted therapies, MRA and/or ASI, not only effectively lower BP but also provide anti-inflammatory and anti-fibrotic benefits and may be effective in reducing both renal and CV risk through BP-lowering and non-BP-mediated effects (*Graphical Abstract*). However, historically, clinical trials for TRH targeted primarily the BP or albuminuria-lowering effects of different interventions such as drug agents and renal denervation, which may limit the adoption in clinical practice.^205–207^ The Blood Pressure Lowering Treatment Trialists’ Collaboration meta-analysis showed that ACEi-based regimens reduced the risk of developing HF, whereas calcium-channel blocker did not despite comparable BP-lowering effects of these regimens.^208^ In the FIDELITY programme, finerenone significantly decreased the risk of developing the HF onset or transitioning to ESKD despite marginal BP-lowering effects in patients with T2D and CKD (apparently high aldosterone state population).^150,158^ These findings emphasize that solely reducing BP may not be a comprehensive approach to preventing the development of life-threatening events. Furthermore, while lowering BP in CKD may potentially reduce CV risk, its impact on CKD progression has yielded conflicting results.^209,210^ As patients with TRH face a dual risk by developing CV and renal events, it is advisable to conduct longer-term studies with hard outcomes such as CV death, HF hospitalization, stroke, MI, and the progression to ESKD or consider a composite cardio-kidney outcome in clinical trials for TRH and CKD.^211^

## Conclusions

**Figure 2:**
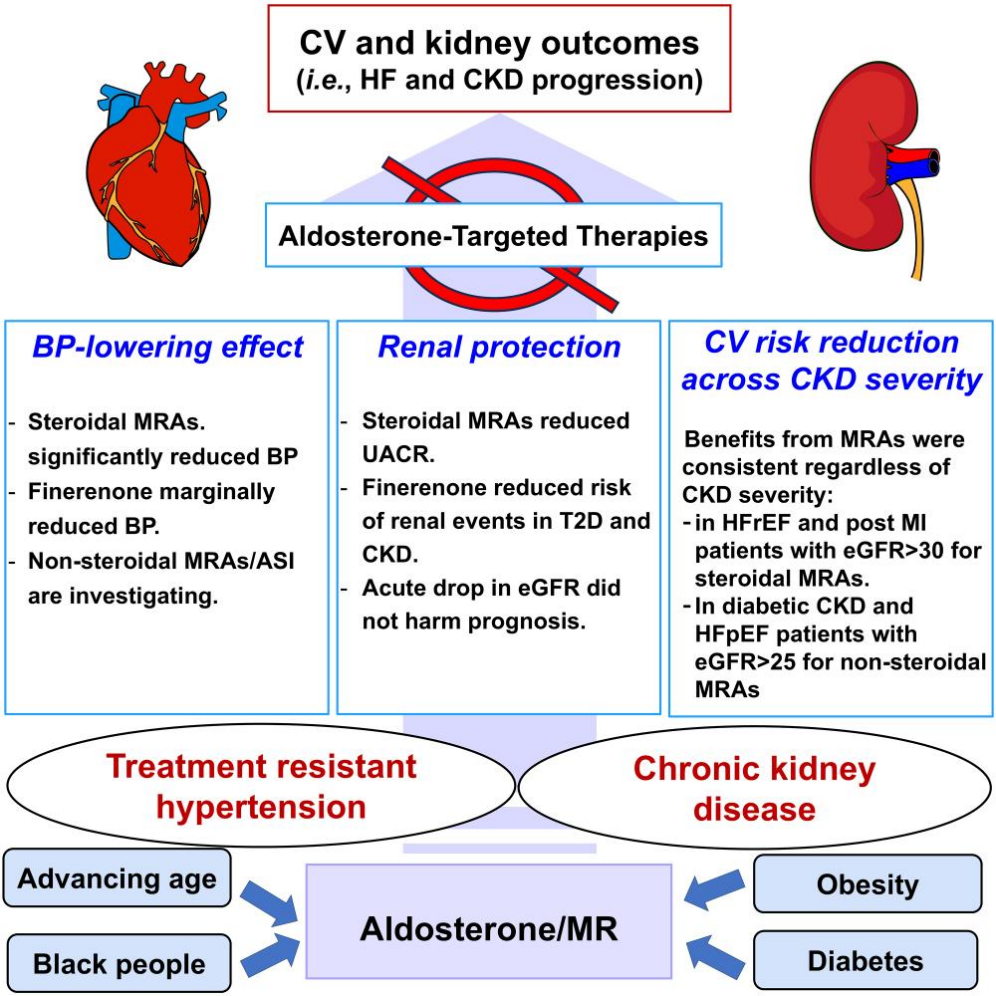
Early implementation of aldosterone-targeted therapies in treatment-resistant hypertension and chronic kidney disease. Clinical trial data show that aldosterone-targeted therapies, particularly MRAs, lower blood pressure, provide renal protection, and reduce the risk of CV events, irrespective of kidney function severity. In people with TRH and/or CKD, characterized by enhanced aldosterone levels and increased MR expression, early implementation of these therapies appears promising. CV, cardiovascular; HF, heart failure; CKD, chronic kidney disease; MRA, mineralocorticoid receptor antagonist; BP, blood pressure; ASI, aldosterone synthase inhibitor; UACR, urine albumin-to-creatinine ratio; T2D, Type II diabetes; eGFR, estimated glomerular filtration rate; HFrEF, heart failure with reduced ejection fraction; MI, myocardial infarction; HFpEF, heart failure with preserved ejection fraction; MR, mineralocorticoid receptor

To effectively reduce CV and renal risk, it is crucial to thoroughly understand the potential mechanisms of development and progression of TRH and CKD. Although routine aldosterone measurements are uncommon in clinical practice due to complex protocols and impact by disease-modifying drugs,^212^ implementing aldosterone/MR-targeted therapy at an early stage of TRH and/or CKD is essential if we wish to avoid its CV and renal consequences (*Figure 2*).

## Declarations

### Data Availability

No data were generated or analysed for this manuscript.
